# Induced Pluripotent Stem Cell-Derived Neural Precursors Improve Memory, Synaptic and Pathological Abnormalities in a Mouse Model of Alzheimer’s Disease

**DOI:** 10.3390/cells10071802

**Published:** 2021-07-16

**Authors:** Enrique Armijo, George Edwards, Andrea Flores, Jorge Vera, Mohammad Shahnawaz, Fabio Moda, Cesar Gonzalez, Magdalena Sanhueza, Claudio Soto

**Affiliations:** 1 Mitchell Center for Alzheimer’s Disease and Related Brain Disorders, Department of Neurology, Mc Govern Medical School, University of Texas, Houston, TX 77030, USA; armijoenrique@hotmail.com (E.A.); George.Edwards@bcm.edu (G.E.); Andrea.Flores@uth.tmc.edu (A.F.); Mohammad.Shahnawaz@uth.tmc.edu (M.S.); fabio.moda@istituto-besta.it (F.M.); cesar.gonzalez@uss.cl (C.G.); 2 Facultad de Medicina, Universidad de los Andes, Av. San Carlos de Apoquindo 2200, Las Condes, Santiago 7550000, Chile; 3 Department of Biology, Faculty of Sciences, University of Chile, Santiago 7800024, Chile; jorgeverab@gmail.com (J.V.); masanhue@uchile.cl (M.S.); 4 Fondazione IRCCS Istituto Neurologico Carlo Besta, Division of Neurology 5 and Neuropathology, 20133 Milan, Italy; 5 Facultad de Medicina y Ciencias, Universidad San Sebastián, Puerto Montt 5480000, Chile

**Keywords:** stem cells, therapy, Alzheimer’s disease, amyloid-beta, tau, inflammation, clinical symptoms

## Abstract

Alzheimer’s disease (AD) is the most common type of dementia in the elderly population. The disease is characterized by progressive memory loss, cerebral atrophy, extensive neuronal loss, synaptic alterations, brain inflammation, extracellular accumulation of amyloid-β (Aβ) plaques, and intracellular accumulation of hyper-phosphorylated tau (p-tau) protein. Many recent clinical trials have failed to show therapeutic benefit, likely because at the time in which patients exhibit clinical symptoms the brain is irreversibly damaged. In recent years, induced pluripotent stem cells (iPSCs) have been suggested as a promising cell therapy to recover brain functionality in neurodegenerative diseases such as AD. To evaluate the potential benefits of iPSCs on AD progression, we stereotaxically injected mouse iPSC-derived neural precursors (iPSC-NPCs) into the hippocampus of aged triple transgenic (3xTg-AD) mice harboring extensive pathological abnormalities typical of AD. Interestingly, iPSC-NPCs transplanted mice showed improved memory, synaptic plasticity, and reduced AD brain pathology, including a reduction of amyloid and tangles deposits. Our findings suggest that iPSC-NPCs might be a useful therapy that could produce benefit at the advanced clinical and pathological stages of AD.

## 1. Introduction

Alzheimer’s disease (AD) is a common, progressive, and devastating neurodegenerative disease characterized by memory impairment and cognitive decline [1]. The most prominent pathological hallmarks of the disease are the extracellular deposition of amyloid β (Aβ) peptides in the form of amyloid plaques and the intracellular accumulation of hyperphosphorylated tau (p-tau) proteins in the form of neurofibrillary tangles (NFTs). Numerous morphological and functional modifications have been associated with these lesions, including dendritic and synaptic alterations, neurodegeneration, as well as the recruitment and activation of microglial and astroglial cells [2].

AD represents a tremendous socio-economic problem due to its devastating nature, monetary cost, and the lack of effective therapies. Therefore, the search for an efficient treatment for this devastating disease is an urgent medical priority [3,4]. At this time, there is no effective therapy against AD. Current therapies (e.g., acetylcholinesterase inhibitors and *N*-methyl-d-aspartate receptor antagonists) only ameliorate the symptoms, but do not delay or halt disease progression [4]. Many of the treatment strategies under development aim at stopping the initiation or slow down the progression of the disease. Unfortunately, several recent clinical trials with compounds targeting the processes that appear to be the triggering events in the pathogenesis, such as amyloid inhibitors, have consistently failed [5]. The most likely reason for these failures is that treatment at the clinical stage of the disease would require recovery of brain functionality, since extensive brain damage occurs before AD can be diagnosed [5].

In recent years, stem cells have received growing attention as a potential regenerative therapy for brain disorders, such as AD. Some success with stem cell therapies has been recently reported in different transgenic animal models of AD as a proof-of-concept [6,7,8]. For these studies, stem cells transplanted into transgenic animals originated from a variety of sources, including the central nervous system (CNS), umbilical cords, amniotic membrane-derived epithelial cells, and mesenchymal stem cells [6,7,8]. In the future, stem cell therapies for neurological disorders likely require the use of patient-specific cells. Immune rejection hinders the use of allogeneic human cells for transplant. Concerns for donor cell rejection could be especially problematic in regard to neurodegenerative diseases such as AD, in which the inflammatory processes underlying this disorder can present an intrinsically hostile environment to any allogenic graft [9,10,11]. Patient-specific stem cells are now possible thanks to the generation of induced pluripotent stem cells (iPSCs) from the somatic cells of the patient, which has been suggested as a new revolutionary step towards personalized medicine [12,13,14]. The use of iPSCs as a source of autologous cells for cell grafting therapies offers the possibility to bypass complications due to immune rejection and the need to use immunosuppressants. Due to their similarities in proliferation, gene expression, the epigenetic status of pluripotent cell-specific genes, and telomerase activity, iPSCs provide an excellent alternative to embryonic stem cells (ESCs) without ethical concerns [15,16]. The application of iPSCs has already advanced rapidly in areas such as the development of cell replacement therapies, disease modeling, and drug screening [12]. iPSC-derived cells have been shown to be effective in animal models of several neurodegenerative diseases including Parkinson’s disease [17,18,19,20], Huntington’s disease [21,22], Multiple Sclerosis [23], and Amyotrophic Lateral Sclerosis [24,25]. Although iPSC-derived neural stem cells (NSCs) and neural precursor cells (NPCs are able to generate neurons, astrocytes, and oligodendrocytes) have been suggested as a potential therapy for AD, and this approach has not been directly tested in an AD model.

In the present study, we investigated how transplanting mouse iPSC-derived neuronal precursors (iPSC-NPCs) into the hippocampus affect cognition, brain activity, synaptic function, Aβ deposition, tau pathology, and the inflammatory response in an aged triple transgenic mouse model of AD (3xTg-AD). We chose this model because these animals display extensive brain damage reminiscent of AD pathology, including the accumulation of amyloid plaques, NFTs, and several markers of neurodegeneration [26,27,28]. Thus, we believe aged 3xTg-AD mimics the condition of a patient with established AD. Our results show that iPSC-NPCs transplantation improved cognitive impairment and boosted long-term potentiation (LTP). Moreover, iPSC-NPCs transplantation reduced AD brain pathology as determined by Aβ deposition, p-tau, and gliosis. Therefore, our findings suggest that iPSC-NPCs might be used as a potential cell therapy for AD and could be extended to treat other neurodegenerative diseases using similar approaches.

## 2. Materials and Methods

### 2.1. Generation of iPSCs from Mouse Tail-Tip Fibroblasts

Mouse tail-tip fibroblasts from adult B6C3/129sv wild type (WT) mice were reprogramed to iPSCs following the protocol described by Takahashi and Yamanaka [15] with minor modifications [29]. Fibroblasts were incubated in retrovirus-containing supernatants overnight (O.N.). After viral infection, cells were re-plated into a six-well plate on irradiated mouse embryonic fibroblasts cells CF-1 (iMEFs, GlobalStem, Rockville, MD, USA) and cultured in standard mouse ESC medium (DMEM) (Invitrogen, Waltham, MA), 15% FBS (Atlanta Biologicals, Flowery Branch, GA), 1x nonessential amino acids (Invitrogen), 1x Glutamax (Invitrogen), 1000 U/mL leukemia inhibitory factor (LIF) (R&D Systems, Minneapolis, MN), and 1x antibiotic and antimitotic stock solution (Invitrogen). The culture medium was replaced daily. Colonies with iPSC-like morphology were picked 3–5 weeks later and expanded on iMEFs in ESC medium. iPSCs were characterized by immunocytochemistry, gene expression analysis, and the ability to differentiate into the 3-germ layers (Figure S1), as previously described [29].

### 2.2. RT-PCR Analysis for Marker Genes

The cDNA was generated using the QuantiTect Reverse Transcription Kit (Qiagen, Hilden, Germany) according to the manufacturer’s procedures. A total of 1 µg of total RNA was used to produce cDNA. RT-PCR was performed for 32 cycles for all markers as follows: Denaturing for 45 s at 94 °C; annealing temperature of 55 °C for 20 s; and extension at 72 °C for 30 s. PCR products were resolved on 2% agarose gel. Primer sequences for mouse ESC genes are listed in Table S1.

### 2.3. Pluripotency Assay of Mouse iPSC Lines

iPSCs were harvested by trypsinization and transferred to low-attachment plates (Corning, Corning, NY, USA) in the mouse ESC medium without LIF. After 3 days, aggregated cells or EB were plated onto gelatin-coated tissue culture dishes and incubated for one week. Samples were fixed and processed for immunofluorescence.

### 2.4. Generation of Mouse NPCs from iPSCs

Derivation of neuropotent self-renewing NPCs was performed as previously described [30]. Mouse iPSCs were harvested, resuspended in neural induction medium (DMEM/F12 and Neurobasal medium) (1:1, Invitrogen), 0.5× N2 supplement (Invitrogen), 1× B27 supplement (Invitrogen), 1× antibiotic, and antimitotic stock solution (Invitrogen), and transferred to 0.1% gelatin-coated plates. Culture medium was replaced every day. After 12 days, cells were harvested by Accutase solution (Millipore, Burlington, MA, USA), resuspended in neural progenitor expansion media (NPEM) (DMEM/F12, 1% N2 supplement, 20 ng/mL EGF and 20 ng/mL bFGF) (R&D Systems) and 1x antibiotic and antimitotic stock solution and transferred to Geltrex LDEV-free reduced growth factor basement membrane matrix- treated (1:100, Invitrogen) dishes. Medium was changed every other day.

### 2.5. GFP Labeling of iPSC-NPCs

iPSC-NPCs were transduced with 17.25 ng of rtTA lentivirus (Stemgent, Cambridge, MA, USA) and 15.65 ng of doxycycline (Dox)-green fluorescent protein (GFP) lentivirus (Stemgent) in NPEM supplemented with 4 μg/mL of polybrene. The culture medium was replaced every day. In vitro, GFP expression was induced using 1 µg/mL of Doxycycline (Sigma, ST Louis, MO). GFP^+^ iPSC-NPCs were then sorted using fluorescence activated cell sorting (FACS) and expanded.

### 2.6. In Vitro Differentiation of Mouse iPSC-NPCs

iPSC-NPCs were differentiated into neurons, astrocytes, or oligodendrocytes in vitro as noted in Figure S3. Culture medium was replaced every 3–4 days in all cases. For differentiation into neurons, iPSC-NPCs were cultured in neural differentiation medium (Neurobasal medium supplemented with 2% of B-27 serum free supplement and 2 mM glutamax-I) for 20 days. For differentiation into astrocytes, iPSC-NPCS were cultured in astrocyte differentiation medium (DMEM supplemented with 1% N-2 supplement, 1% FBS, and 2mM Glutamax-I) for 10 days. For differentiation into oligodendrocytes, iPSC-NPCS were cultured in oligodendrocyte differentiation medium (Neurobasal medium supplemented with 2% of B-27 serum free supplement, 2 mM glutamax-I, and 30ng/mL of 3,3′,5-Triiodo-l-thyronine (T3) (Sigma-Aldrich, St Louis, MO, USA) for 21 days.

### 2.7. Flow Cytometry Analysis

iPSC-NPCs were detached, counted, and fixed for 20 min with BD Cytofix Fixation buffer (BD Bioscience, Franklin Lakes, NJ, USA) at room temperature (RT). Cells were permeabilized for 30 min on ice using BD Phosflow Perm Buffer III (BD Bioscience) and resuspended in PBS with 2% FBS at a concentration of 10^6^ cells per 100 μL. Antibodies at the appropriate dilution were added to the cells, and the mixture was incubated for 30 min in the dark. Cell suspensions were stained using the fluorochrome-conjugated monoclonal antibodies against Nestin AlexaFluor 647, E-Cadherin phycoerythrin (PE), DCX PE, GFAP AlexaFluor 647, and Oct4 AlexaFluor 647 (BD Bioscience). Data were collected on a BD LSR II flow cytometer (BD Biosciences) and analyzed using either FACS Diva (BD Biosciences) or FCS Express 5 Flow Cytometry software (De Novo Software, v5.0).

### 2.8. Transgenic Mice

3xTg-AD (Jackson Laboratory, Bar Harbor, ME, USA) is a transgenic mouse model for AD that expresses a chimeric mouse/human amyloid precursor protein (APP) containing the Swedish mutation, the M146V mutant form of human presenilin 1 (PS1), and a mutant form of tau (P301L) transgene. This mouse model develops AD-related abnormalities, including memory impairment, Aβ plaques, p-tau, and inflammation [27]. Non-transgenic control mice were maintained by crossing WT hybrid B6C3 mice with each other. All groups contained male and female mice with approximately 62–67% females. For this study, we used 17-month-old 3xTg-AD mice that exhibited substantial pathological and behavioral changes associated with AD. All animal procedures described in this article were in agreement with the regulations of the Center for Laboratory Animal Medicine and Care (CLAMC) and Animal Welfare Committee (AWC) of the University of Texas Medical School at Houston.

### 2.9. Surgical Procedures and Cell Transplantation

3xTg-AD mice were deeply anesthetized with isofluorane and injected bilaterally in the hippocampus with 5 × 10^5^ iPSC-NPCs in 10 μL of PBS or the vehicle control (PBS). The head was shaved, sterilized, incised, and a small hole was drilled in the skull. Using a Hamilton 33-gauge 10-µL syringe (Hamilton Company, Reno, NV, USA), iPSC-NPCs or vehicle control were stereotaxically injected bilaterally using the following coordinates as measured from bregma: Anteroposterior (AP), −2.0 mm; mediolateral (ML), +/−2.0 mm; dorsoventral (DV), −1.5 mm, −2.1 mm, and −2.8 mm from the skull. ~2.5 × 10^5^ cells (5 µL), ~1.25 × 10^5^ cells (2.5 µL), and ~1.25 × 10^5^ cells (2.5 µL) were infused into each site of the hippocampus. To induce GFP expression in iPSC-NPCs, both groups of mice were treated with food pellets containing 200 mg/kg of Doxycycline (Dox) ad libitum from the first day post-injection until sacrificed.

### 2.10. Object Location Task (OLT)

Animals were examined over a 5-day period using the OLT to assess spatial memory and discrimination. This test is based on the spontaneous tendency of rodents to spend more time exploring a novel object than a familiar object and to also recognize when an object has been relocated. First, the mouse was acclimated to the environment by exploring an empty Plexiglas box (42 cm × 42 cm) for 2 trials (5 min, then 10 min) on day 1, followed by one trial for 10 min on days 2–3. The mouse was then introduced to 2 identical objects (circles or blocks) on day 4 for 10 min. On day 5, the rodent was exposed for 5 min to the familiarized objects with one displaced to a new location. Mice were assayed before injection and 1 month post-injection while alternating the objects pseudo-randomly. A discrimination ratio was obtained at the final testing by dividing the novel quadrant time by the total time of both the novel and familiar quadrants. A discrimination ratio higher than 50% demonstrates intact cognitive abilities, and a discrimination ratio lower than 50% demonstrates impaired cognitive abilities. Mice spending less than 10 s combined with the objects or demonstrating motor impairments were not used due to a lack of participation. The experimenter was blind to animal genotype and treatment. Trials were recorded and analyzed by TopScan 2.0 software.

### 2.11. Barnes Maze Assessment

The Barnes maze was performed as previously [31,32]. The maze is a large circular platform measuring 1.22 m in diameter with 40 holes lining the outside of the platform and is surrounded by black curtains with fixed geometric shapes. To ensure participation, we utilized a beeping sound that played during the trial. Mice were assayed 2 months post-injection and trained for 5 days with 4 trials per day. Briefly, on day 1, the mice were familiarized and adapted to the task. Days 2–5 comprised of training for spatial acquisition, where the animal was placed in the center of the platform and allowed to explore the maze for a maximum of 3 min. The primary latency to the escape hole was averaged over training trials in blocked days. Long-term memory, which consisted of 1 trial, was assessed at day 12. The experimenter was blind to animal genotype and treatment. Any animal demonstrating motor impairments by visual inspection was not used in analyses. Trials were recorded and analyzed by TopScan 2.0 software.

### 2.12. Immunocytochemistry

Mouse iPSC, embryoid bodies (EB), and iPSC-NPCs were fixed in Bouin’s solution (Sigma-Aldrich) for 15 min at 4 °C, washed with 5 mM Tris-HCl buffer (pH 7.8), and stored in the same buffer at 4 °C until further processing. Samples were incubated in the primary antibodies diluted in Tris-carrageenan-triton solution (TCT) (Tris-HCl buffer pH 7.8, 0.7% λ-carrageenan (Sigma), and triton 0.5% X-100 (Sigma)) O.N. at RT using the primary antibodies against: Oct4 (Abcam, Cambridge, UK, 1:100), Sox2 (Abcam, 1:100), SSEA-1 (Developmental Studies Hybridoma Bank (DSHB), 1:10), E-Cadherin (BD Bioscience, 1:15), Brachyury (Abcam, 1:100), α-fetoprotein (Abcam, 1:100), MAP2 (Millipore, 1:500), Nestin (DSHB, 1:10), Vimentin (Abcam, 1:50), Musashi (Abcam, 1:200), Doublecortin (DCX) (Abcam, 1:200), Olig1 (Millipore, 1:200), glial fibrillary acidic protein (GFAP) (Abcam, 1:500), and 2′,3′-Cyclic-nucleotide 3′-phosphodiesterase (Abcam, 1:200). Samples were subsequently incubated for 1 h at RT with Alexa Fluor-labeled secondary antibodies (Invitrogen) diluted at 1:500 in TCT. Finally, the cells were mounted in VECTASHIELD HardSet Mounting Medium (Vector laboratories, Burlingame, CA, USA) with 4′,6-diamidino-2-phenylindole (DAPI). Images were acquired with a Leica DMI 6000B microscope. For alkaline phosphatase (AP) staining, iPSCs were stained using the AP staining kit (Stemgent) according to the manufacturer’s instructions. Briefly, cells were fixed at RT for 1 to 2 min, washed, and incubated for 30 min with freshly prepared AP staining solution. The reaction was stopped by aspirating the AP staining solution and washing with PBS.

### 2.13. Immunohistochemistry

Male and female mice were sacrificed at 19 months old by CO_2_ inhalation and perfused with cold 1x PBS containing 5mM EDTA. The left brain hemisphere was snap-frozen in liquid nitrogen and stored at −80 °C until use for biochemical studies. To analyze the distribution of iPSC-NPCs, the right brain hemisphere was fixed in cold 4% paraformaldehyde fixative overnight. Fixed brains were immersed in 30% sucrose and sectioned at 20-µm intervals using a cryostat. Five slices (one every ten) per animal were processed for immunohistochemistry. Sections were hydrated in Tris-HCl buffer (pH 7.8), blocked, permeabilized, and incubated overnight in TCT buffer with one of the primary antibodies against enhanced green fluorescent protein (eGFP; Invitrogen, 1:200), Nestin (DSHB, 1:10), GFAP (Millipore, 1:500), DCX (Abcam, 1:200), and CNPase (Abcam, 1:200). The primary antibodies were revealed after incubating for 1 h at RT with Alexa Fluor-labeled secondary antibodies (Invitrogen) diluted at 1:500 in TCT. Finally, the cells were mounted in VECTASHIELD HardSet Mounting Medium (Vector laboratories) with DAPI. Images were acquired with a Leica DMI 6000B microscope.

For studies focused on AD pathology, the right brain hemispheres were fixed in Carnoy’s solution (60% ethanol, 30% chloroform, and 10% glacial acetic acid) O.N. and dehydrated for paraffin inclusion. Five slices (one every ten) per animal were processed for immunohistochemistry. Briefly, sections were deparaffinized and the endogenous peroxidase activity was blocked with 6% H2O2 for 15 min. Sections were incubated O.N. at RT with the AT8 anti-p-tau antibody (ThermoFisher, 1:200, epitope phosphorylated Ser-202 and Thr-205) or anti-GFAP (1:200, Millipore), or sections were treated in 80% formic acid (FA) for 30 min and incubated O.N. at RT with the 4G8 anti-Aβ antibody (Covance, Pribceton, NJ, 1:1000, epitope 17-24). The primary antibodies were revealed with a HRP-linked secondary sheep anti-mouse antibody (GE Healthcare, Chicago, IL, USA, 1:500). Peroxidase reaction was visualized using a DAB Kit (Vector) following the manufacturer’s instructions. Finally, sections were dehydrated in graded ethanol, cleared in xylene, and cover-slipped with DPX mounting medium (Innogenix, Amityville, NY, USA). Thioflavin-S (ThS) staining was performed by incubating tissue slices with a ThS (Sigma) solution (0.1% in 50% ethanol) for 15 min after deparaffinization.

### 2.14. Image Analyses

For quantification, brain slices were examined under a DMI6000B microscope and image analysis was performed using the ImageJ software (National Institutes of Health, Bethesda, MD, USA). 4G8, AT8, and GFAP burden were defined as the antibody labeled area in each tissue slice per total area analyzed (hippocampal and cortical areas only), averaged per slide, and expressed as a percentage, as done previously [31,32].

### 2.15. Electrophysiological Recordings in 3xTg-AD Brain Slices

Transverse hippocampal slices of 400 µm were prepared from 19-month-old 3xTg-AD mice injected with iPSC-NPCs or PBS in ice-cold dissection solution containing (in mM): 125 NaCl, 2.6 KCl, 10 MgCl_2_, 0.5 CaCl_2_, 26 NaNCO_3_, 1.23 NaH_2_PO_4_, and 10 d-glucose (equilibrated with 95% O_2_ and 5% CO_2_), pH 7.3. Slices were transferred to a submersion-type incubation chamber in artificial cerebrospinal fluid (CSF) containing (in mM): 125 NaCl, 2.6 KCl, 1 MgCl_2_, 2 CaCl_2_, 26 NaNCO_3_, 1.23 NaH_2_PO_4_, and 10 d-glucose (95% O_2_ and 5% CO_2_), pH 7.3. Slices were allowed to recover for a minimum of 1 h at RT before recording. Slices were mounted on an upright BX51 microscope (Olympus, Shinjuku City, Tokyo, Japan) and were continuously superfused with oxygenated artificial CSF at a rate of 2 mL/min at 30 °C. Excitatory postsynaptic field potential (fEPSP) recordings were conducted to monitor synaptic transmission and plasticity. The experimental configuration consisted of three electrodes placed in the CA1 region of the hippocampus. A borosilicate-glass electrode (3–5 MΩ) was positioned in stratum radiatum for field potential recordings and a similar electrode was used for the synaptic stimulation of Schaffer collaterals at ~200 µm from the recording electrode. In addition, a tungsten electrode (5 MΩ) was used to stimulate a different synaptic pathway that served as a control pathway in plasticity experiments.

After reaching a stable transmission baseline (5–10 min), input-output (I-O) curves were obtained by applying stimuli of increasing intensities (10–80 µA in 10 µA steps, 2 recordings per stimulus amplitude) until signal saturation or population spike generation. Paired-pulse facilitation was explored using 20, 60, 100, 200, and 500 ms intervals between the first and second stimuli. LTP was induced after 10 min of stable basal recording using two sequential stimulation paradigms. First, a weak-intensity theta-burst stimulation (TBS) protocol consisting of 3 bursts (each of 4 pulses at 100 Hz) separated by 200 ms was applied. This was followed 20 min later by a high-frequency stimulation (HFS) protocol comprising 4 tetani (100 pulses at 100 Hz, each) separated by 20 s. To evaluate the effect on synaptic strength produced by each type of stimulation, the fEPSP slope was normalized to basal levels and the changes were calculated relative to the unstimulated control pathway. For paired-pulse facilitation and LTP experiments, the intensity of presynaptic stimulation was set to produce 50–60% of the maximal response. Data was collected using a Multiclamp 700B amplifier, filtered at 10 kHz, digitized at 5 kHz, and stored in a PC using pClamp 10 software (Molecular Devices, San Jose, CA, USA) and analyzed offline using Igor Pro 6.2 software (WaveMetrics, Tigar, OR, USA).

### 2.16. Amyloid-Beta (1-x) ELISA

Left brain hemispheres were homogenized at 10% (*w/v*) in ice-cold PBS containing a cocktail of protease inhibitors (Roche Diagnostics, Risch-Rotkreuz, Switzerland). Resulting homogenates were stored at −80 °C until use. In order to measure the amount of soluble and insoluble Aβ, 200 μL of aliquots of each sample were centrifuged at 100,000× *g* for 1 h at 4 °C using a L100K ultracentrifuge (Beckman-Coulter, Brea, CA, USA). Supernatants were recovered and saved as “PBS fractions” or “soluble Aβ fraction” and pellets were resuspended in 200 μL of 70% FA and centrifuged for 30 min using the same temperature and speed previously described. Resulting supernatants (“FA fractions” or “insoluble Aβ fraction”) were diluted 20 times in 1 M Tris buffer (pH 11) to adjust pH. All resulting samples were stored at −80 °C until measured by a solid phase sandwich ELISA for the determination of a total human Aβ (1-x) (IBL America, Spring lake Park, MN, USA).

### 2.17. Statistical Analysis

Graphs were expressed as means ± standard error of the mean (S.E.M.). One-way and repeated measures two-way analysis of variance (ANOVA) followed by a post-hoc Tukey’s multiple comparisons test and Bonferroni post-test were used to analyze differences among groups, respectively. Within subject OLT testing was analyzed by paired two-tailed *t*-test. Student’s *t*-test was used to compare 3xTg-AD and WT OLT performance and immunohistochemistry and biochemical aggregate burden in injected and non-injected animals. Statistical differences for all tests were considered significant at the *p* < 0.05 level. Statistical analysis was performed using GraphPad Prism 5.0 software (GraphPad Software Inc., San Diego, CA, USA).

## 3. Results

### 3.1. Generation of iPSCs and Derivation of Neuronal Precursors from iPSCs

To study whether iPSC-NPCs transplantation improves AD pathology and cognition in a mouse model, we first reprogrammed WT mouse tail-tip fibroblasts to generate iPSCs. Cells were characterized by immunocytochemistry using various markers for embryonic stem cells, RT-PCR to analyze the level of expression of stem cell markers, and a pluripotency assay to show that iPSCs can originate cells from the three germ layers (Figure S1). Subsequently, iPSCs were differentiated into a monolayer of self-renewing NPCs, called iPSC-NPCs, which were characterized by immunocytochemistry and flow cytometry (Figure S2) as well as by their ability to give rise to neurons, astrocytes, and oligodendrocytes (Figure S3). In order to visualize the iPSC-NPCs cells after in vivo injection, we labeled them with GFP under the control of a doxocycline-activated promotor (Figure S4).

### 3.2. iPSC-NPCs Survive, Migrate, Incorporate, and Differentiate in an Aged 3xTg-AD Mouse Brain

**Figure 1:**
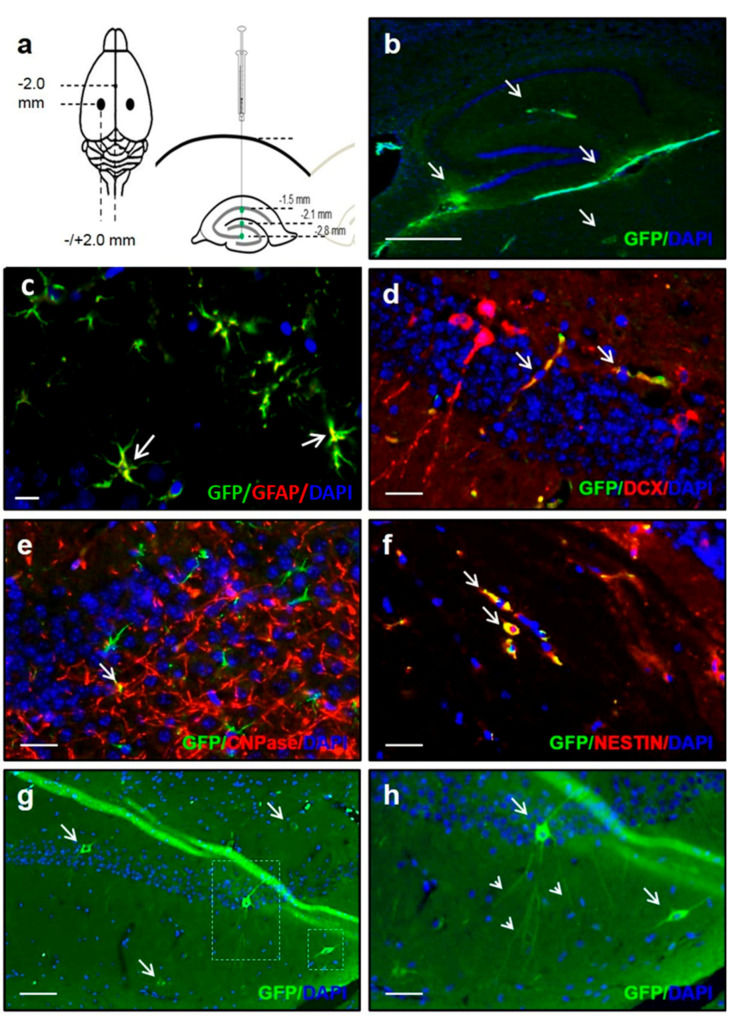
iPSC-NPCs survive, migrate, and differentiate in the 3xTg-AD mice brain. (**a**) iPSC-NPCs were stereotaxically injected bilaterally using the following coordinates as measured from bregma: Anteroposterior (AP), –2.0 mm; mediolateral (ML), +/−2.0 mm; dorsoventral (DV), −1.5 mm, −2.1 mm, and −2.8 mm from the skull. ~2.5 × 10^5^ cells (5 µL), ~1.25 × 10^5^ cells (2.5 µL), and ~1.25 × 10^5^ cells (2.5 µL) were infused into each site of the hippocampus. Two months after delivery, 19-month-old 3xTgAD mice were sacrificed and the engraftment, migration, and differentiation of mouse iPSC-NPCs in the brain were examined. (**b**) A limited migration of iPSC-NPCs around the hippocampal area and thalamus was observed. Scale bar: 300 μm. Engrafted iPSC-NPCs differentiated into all three lineages: Astrocytes (**c**), neurons (**d**), and oligodendrocytes (**e**). (**f**) Some GFP-positive cells co-expressing the Nestin marker remained as NPCs. Scale bar (**c**–**f**): 25 μm. (**g**,**h**) GFP-positive cells that exhibited a pyramidal neuronal morphology and GFP-labeled dendritic spines were also occasionally observed. Scale bar: 100 μm. (**h**) Magnification of the areas marked in (**g**) with a 50-μm scale bar. White arrows in some panels highlight cells that are positive for the respective markers.

Next, we stereotaxically delivered 5 × 10^5^ iPSC-NPCs to both hippocampal hemispheres of 17-month-old 3xTg-AD mice with advanced plaque and tangle pathology. Injection was done at three different depths of the hippocampal formation to increase the number of cells administered (Figure 1a). As a control, age-matched 3xTg-AD mice received similar injections with an equivalent volume of PBS (vehicle). Two months after delivery and upon doxycycline treatment, we observed a limited migration of GFP-positive iPSC-NPCs around the hippocampal area and thalamus (Figure 1b, arrows). Engrafted iPSC-NPCs were observed to differentiate into the three neural lineages in a manner similar to reports for fetal NSC [33,34,35], namely: Astrocytes co-expressing GFP and GFAP (30.4% ± 3.8; Figure 1c); neurons co-expressing GFP and the early neuronal marker DCX (4.8% ± 2.0; Figure 1d); and oligodendrocytes co-expressing GFP and 2′,3′-cyclic nucleotide-3′-phosphodiesterase (CNPase; 1.4% ± 0.6; Figure 1e). A small proportion of the GFP-positive iPSC-NPCs (1.2% ± 1.2) remained NPCs, as indicated by the co-expression of the Nestin marker (Figure 1f). Additionally, few GFP-positive cells exhibiting pyramidal neuronal morphology and GFP-labeled proximal axons were occasionally observed (Figure 1g,h). Importantly, there was no tumor formation in any of the iPSC-NPCs-injected mice after 2 months.

### 3.3. 3xTg-AD Mice Injected with iPSC-NPCs Exhibit Improved Learning and Memory

**Figure 2:**
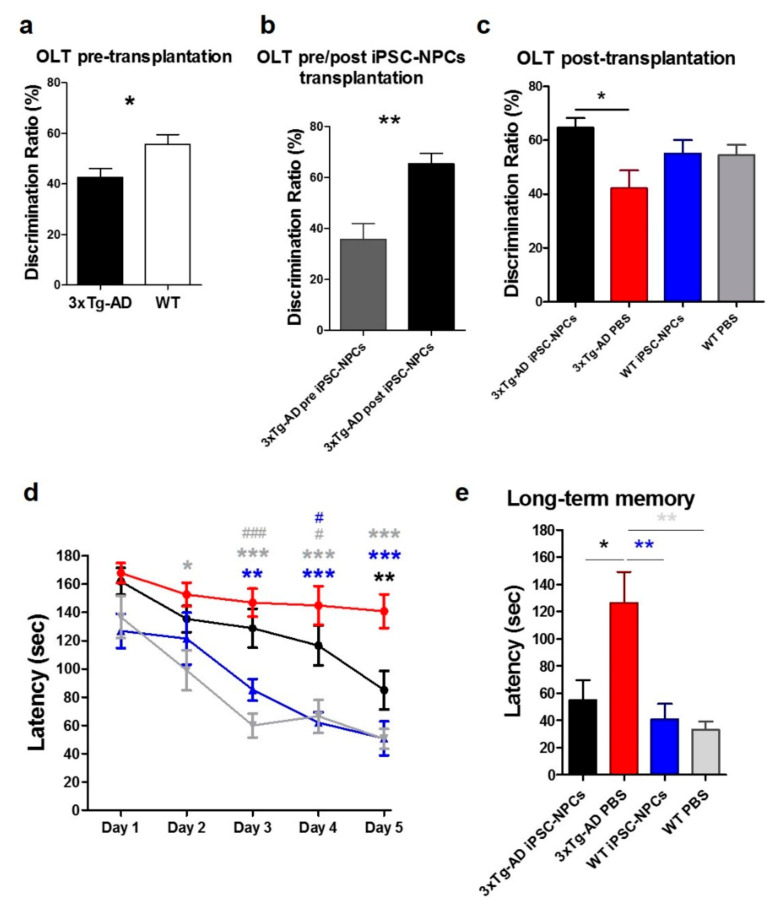
3xTg-AD mice transplanted with iPSC-NPCs showed an improved performance in the object location task (OLT) and Barnes Maze test. Aged 3xTg-AD mice and aged-matched WT controls were tested in the OLT using circle or block objects in different quadrants pseudo-randomly before injection at 17 months and after injection at 18 months. Animals that did not explore the objects ≥ 10 sec or exhibited any motor impairment were removed from analysis. (**a**) 17-month-old 3xTg-AD mice (*n* = 23) demonstrated a significant impairment in spatial abilities compared to aged-matched WT mice (*n* = 14) (unpaired *t*-test, * *p* < 0.05). (**b**) Within subject testing of iPSC-NPCs injected 3xTg-AD mice (*n* = 7) suggested a significant improvement in performance from prior testing to iPSC-NPCs injection (paired *t*-test, ** *p* < 0.01). (**c**) One month after injection, iPSC-NPCs-injected 3xTg-AD mice spent significantly more time in the novel quadrant than PBS-injected 3xTg-AD mice; in addition, iPSC-NPCs-injected 3xTg-AD mice performed on par with WT controls (*n* = 7–8/group; one-way ANOVA with Tukey’s multiple comparison test, *p* < 0.05; ** *p* < 0.01). The same aged 3xTg-AD or WT controls mice groups were assayed two months post-injection at approximately 19 months of age using the Barnes maze test (*n* = 6–12/group). (**d**) PBS-injected 3xTg-AD mice exhibited longer latency to the escape hole over time compared to iPSC-NPCs-injected 3xTg-AD mice (** *p* < 0.01), iPSC-NPCs-injected WT mice (** *p* < 0.01; *** *p* < 0.001), and PBS injected-WT mice (* *p* < 0.05; *** *p* < 0.001) control groups. WT PBS (^#^ *p* < 0.05; ^###^ *p* < 0.001) and WT iPSC-NPCs mice (^#^ *p* < 0.05) learned the task faster than 3xTg-AD iPSC-NPCs mice however, there was no difference in performance at final testing (two-way ANOVA with Bonferroni post-test, *F*_(3,31)_ = 16.59; *p* < 0.0001). (**e**) 3xTg-AD iPSC-NPCs mice demonstrated shorter latency to the escape hole in long-term memory assessment compared to 3xTg-AD PBS mice (* *p* < 0.05). 3xTg-AD iPSC-NPCs mice also performed on par with WT control groups. As expected, WT iPSC-NPCs (** *p* < 0.01) and WT PBS mice (** *p* < 0.01) performed better than 3xTg-AD PBS mice in long-term memory (one-way ANOVA with Tukey’s multiple comparison test, *F*_(3,31)_ = 6.793, *p* < 0.01). Data corresponds to the means ± SEM.

Progressive cognitive decline is a hallmark characteristic of AD [2], which can also be observed in the 3xTg-AD mice model [36]. To examine whether iPSC-NPC transplantation improved spatial learning and memory, 3xTg-AD mice were analyzed before and after injection using the object location task (OLT) and the Barnes maze test, respectively (Figure 2). In comparison to aged-matched WT mice (*n* = 14) in the OLT, 17-month-old 3xTg-AD mice (*n* = 23) demonstrated a significant impairment in spatial abilities by spending more time in the familiar quadrant than the novel quadrant prior to treatment (*F*_(1,35)_ = 5.89, *p* < 0.05; Figure 2a). Thereafter, 3xTg-AD and WT animals were segregated into their respective groups being treated with iPSC-NPCs or PBS (*n* = 7–8/group) and assessed in the OLT with different objects and quadrants. Within subject testing for 3xTg-AD mice injected with iPSC-NPCs (*n* = 7) suggested a significant improvement in an OLT performance between the test prior to injection and the test 1 month post injection (*p* < 0.01, Figure 2b). There was no significant difference in OLT performance pre- and post-injection among the 3xTg-PBS, WT-NPCs, and WT-PBS control groups, respectively (*p* > 0.05, ns, data not shown). Intriguingly, one-way ANOVA demonstrated that the 3xTg-AD mice injected with iPSC-NPCs spent more time in the novel quadrant than 3xTg-AD PBS mice (Figure 2c). In addition, 3xTg-AD mice injected with iPSC-NPCs performed on par with WT controls (Figure 2c) (*F*_(3,25)_ = 3.71, *p* < 0.05).

The same groups (*n* = 6–12/group) were assayed 2 months post-injection using Barnes maze. Learning was apparent in all groups, as performance improved over the trials (*F*_(4,124)_ = 23.03, *p* < 0.0001) (Figure 2d). However, 3xTg-AD mice treated with PBS demonstrated the most difficulty in discovering the hidden escape hole compared to 3xTg-AD treated with iPSC-NPCs and both WT mice groups (*p* < 0.0001, Figure 2d). WT iPSC-NPCs and WT PBS mice seemed to learn the task quicker than the 3xTg-AD iPSC-NPCs group, yet the 3xTg-AD iPSC-NPCs mice ultimately performed on par compared to both WT groups with significantly shorter latency than their 3xTg-AD PBS-injected mice counterpart by the fifth day. There was no difference in performance between WT iPSC-NPCs and WT PBS mice. We also analyzed long-term memory measured as the latency to the escape hole 7 days after the final trial (Figure 2e). Astonishingly, 3xTg-AD iPSC-NPCs mice were able to recall the escape hole location even after 7 days of the last trial. 3xTg-AD iPSC-NPCs mice and WT controls spent less time to find the escape hole than 3xTg-AD mice treated with PBS and even performed comparably to WT iPSC-NPCs and PBS controls (*F*_(3,31)_ = 6.793, *p* < 0.01, Figure 2e). Taken together, these results demonstrate that the injection of iPSC-NPCs significantly improved the cognitive performance in aged 3xTg-AD mice to levels comparable to WT mice without any adverse effects from the iPSC-NPCs injection itself.

### 3.4. 3xTg-AD Mice Injected with iPSC-NPCs Improved Brain Activity and Synaptic Plasticity

**Figure 3:**
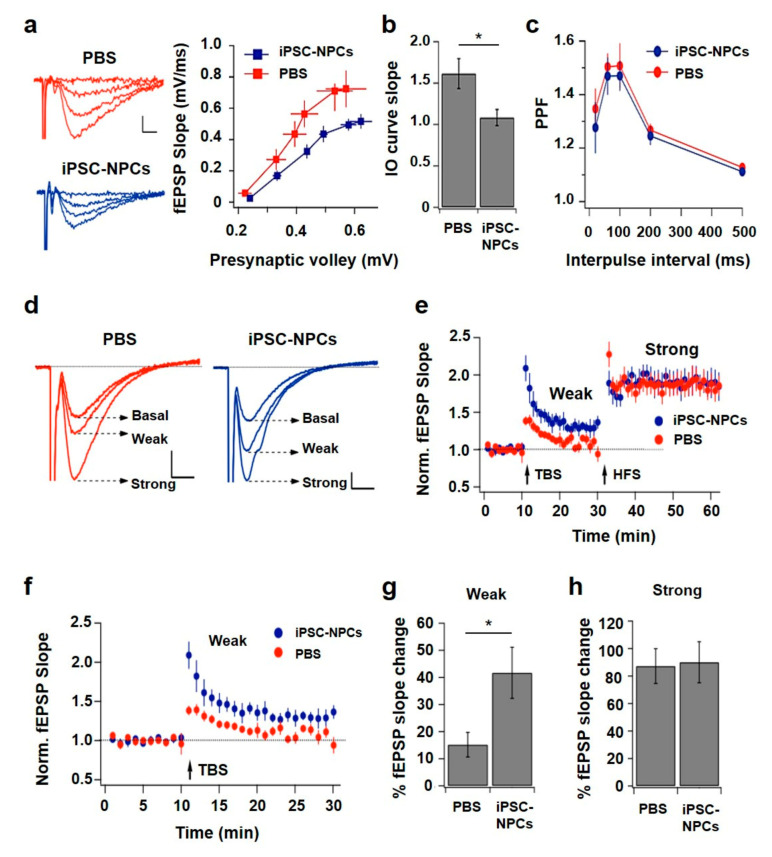
Reduced synaptic strength and lower threshold for LTP induction in 3xTg-AD mice transplanted with iPSC-NPCs. (**a**) Superimposed representative fEPSP recordings for increasing stimulus amplitudes (left) and average input-output (I-O) curves (right) from brain slices of 3xTg-AD mice injected with PBS or iPSC-NPCs (*n* = 22 and 13 slices, respectively). Calibration: 0.5 mV, 2 ms. (**b**) Average slope from the I-O curves shown in (**a**), with 1.61 ± 0.18 (1/ms) and 1.08 ± 0.10 (1/ms) for PBS and iPSC-NPCs-injected mice, respectively (*p* = 0.006, *t*-test, * *p* < 0.05). (**c**) Paired-pulse facilitation at different inter-pulse intervals (20–500 ms) for the two groups of mice (*n* = 6 slices for PBS and 5 for iPSC-NPCs). (**d**) Weak (TBS) and strong (HFS) LTP-induction protocols were subsequently applied to slices from PBS- and iPSC-NPCs-injected mice. The superimposed traces represent the averages of recorded fEPSP in basal conditions and after weak and strong stimulation for PBS (left) and iPSC-NP-treated mice. Calibration 0.2 mV, 5 ms. (**e**) Average graph of normalized fEPSP slope throughout the experiments performed in the two groups of mice (*n* = 9 and 6 slices for PBS and iPSC-NPCs respectively). Data was calculated with respect to an unstimulated control pathway. (**f**) Zoom of the graph in (**e**), to compare the changes produced by the weak stimulation. (**g**) Quantification of percent synaptic potentiation induced by the weak protocol (15.2 ± 4.5% and 41.7 ± 9.4% for PBS and iPSC-NPCs, respectively; *p* = 0.007, *t*-test, * *p* < 0.05). Values are the average for 20 min after induction. (**h**) Same as (**g**), for strong stimulation (87.2 ± 12.5 vs 90.0 ± 14.9, for PBS and iPSC-NP, respectively; *p* = 0.445, *t* test). Average for 30 min after induction.

Synaptic dysfunction closely correlates with memory and cognitive impairments in AD, suggesting that synaptic changes are crucial for AD pathogenesis [37,38,39]. NMDA-receptor dependent LTP of glutamatergic synapses is a major cellular model of synaptic plasticity underlying learning and memory processes in the hippocampus [40]. For this reason, we assessed the effect of injecting iPSC-NPCs on synaptic function by conducting electrophysiological experiments in brain slices from the 3xTg-AD mice injected with iPSC-NPCs or PBS. We recorded fEPSP evoked in CA1 stratum radiatum after increasing the amplitude for the stimulation of the Schaffer collaterals in the brain slices (interleaved experiments; Figure 3a left). Intriguingly, the comparison of I-O curves indicated that iPSC-NPCs transplanted into 3xTg-AD mice resulted in reduced basal transmission compared to the PBS injected 3xTg-AD mice; i.e., for a similar discharge of presynaptic axons, the response is lower in the test group (Figure 3a right). Quantification of this difference demonstrated that iPSC-NPCs-transplanted mice (1.08 ± 0.10 (1/ms)) presented a significantly lower I-O curve slope compared to PBS-injected mice (1.61 ± 0.18 (1/ms); *p* = 0.006) (Figure 3b).

To evaluate if the reduction in synaptic strength was due to a pre- or post-synaptic mechanism, we studied the paired-pulse facilitation of fEPSP in brain slices from iPSC-NPCs and PBS-injected 3xTg-AD mice. The two groups of mice displayed comparable synaptic facilitation with a peak ratio near 1.5 in the range of 60–100 ms of interpulse interval (Figure 3c). The similarity of the curves obtained in both conditions suggested that the injection of iPSC-NPCs did not change the mechanism of presynaptic facilitation.

Therefore, our interpretation is that the synaptic strength reduction was not caused by a decrease in release probability at individual synaptic contacts. Postsynaptic changes and/or a decrease in the number of connections may underlie the observed difference in basal transmission. To investigate if synaptic plasticity was altered in CA1-CA3 connections of the iPSC-NPCs transplanted mice, we assessed the ability of these synapses to undergo LTP in brain slices. We examined synaptic potentiation using a weak stimulation protocol (theta-burst stimulation or TBS) and a strong protocol (high frequency stimulation or HFS; Figure 3d). In slices from PBS-injected 3xTg-AD mice, the weak stimulation produced a slight and transient increase in synaptic transmission. This short-term potentiation declined rapidly reaching baseline in a few min. In contrast, the strong protocol potentiated transmission to nearly twice the baseline and effect was stable for at least the next 30 min (Figure 3e). Interestingly, when studying the synaptic changes generated by the two stimulation protocols in slices from the 3xTgAD mice that received the iPSC-NPCs, we observed that TBS induced a potentiation that lasted at least until the application of the second stimulus (Figure 3f). In these slices, subsequent HFS strongly potentiated transmission, closely resembling what was observed in slices from PBS-injected 3xTg-AD mice. Average percent potentiation induced by TBS in slices from iPSC-NPCs-transplanted 3xTg-AD mice was significantly higher than for PBS-injected 3xTg-AD mice (15.2 ± 4.5% and 41.7 ± 9.4% for PBS and iPSC-NPCs, respectively; *p* = 0.007) (Figure 3g). In contrast, LTP induced by HFS was similar in both groups (Figure 3h). In summary, hippocampal brain slices from 3xTgAD mice treated with iPSC-NPCs displayed reduced basal transmission, but an increase in synaptic potentiation produced by a weak LTP-inducing protocol. On the other hand, LTP generated by a stronger stimulation was comparable between groups.

### 3.5. 3xTg-AD Mice Transplanted with iPSC-NPCs Showed a Reduction in AD Brain Pathology

**Figure 4:**
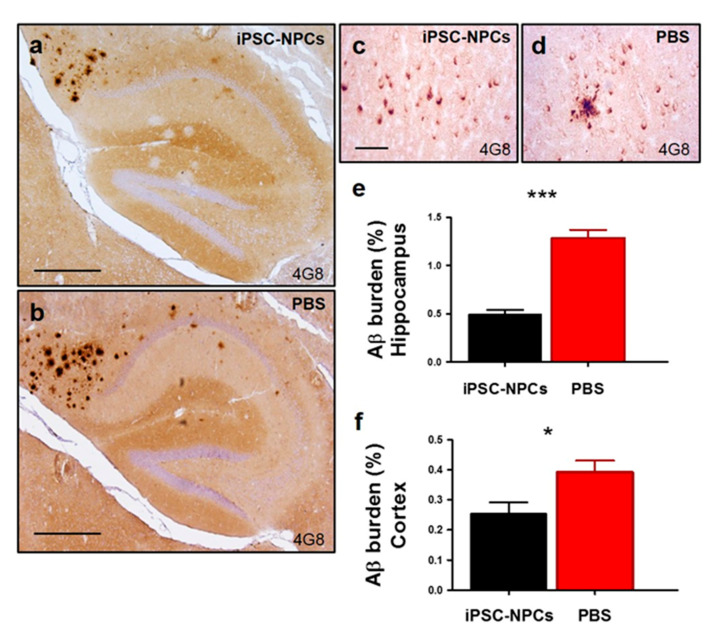
Reduction of Aβ pathology in 3xTg-AD mice transplanted with iPSC-NPCs. (**a**–**d**) Brains from 3xTg-AD iPSC-NPCs and PBS-injected mice were analyzed by Aβ immunostaining (4G8 antibody, which recognizes amino acid residue 17–24 of Aβ). Representative pictures of the amyloid deposits showed a decrease in the hippocampus and cortex of 3xTg-AD mice injected with iPSC-NPCs (**a**,**c**) compared with PBS-injected mice (**b**,**d**). Light microscopic images of 4G8-immunoreactivity were counterstained with hematoxylin in the hippocampus (**a**,**b**) and cortex (**c**,**d**) of PBS and iPSC-NPCs transplanted mice. Scale bar (**a**,**b**): 300 μm; (**c**,**d**): 25 μm. Image analysis was done to estimate the amyloid burden in the hippocampus (**e**) and cortex (**f**). 4G8 burden was higher in 3xTg-AD PBS mice compared to 3xTg-AD iPSC-NPCs mice in the hippocampus (*** *p* < 0.001) and cortex (* *p* < 0.05) by student *t*-test (*n* = 7–8/group). Data corresponds to the means ± SEM.

To determine whether injection of iPSC-NPCs modified Aβ deposition in the 3xTg mouse model, we analyzed histological Aβ burden and ThS-positive amyloid accumulation, as well as biochemical quantification of insoluble Aβ in the hippocampus and cortex—both vital regions affected by AD. Histological analysis of Aβ burden using 4G8 antibody demonstrated a significant reduction in the hippocampus (Figure 4a,b,e; fold change (FC): 2.61) and the cerebral cortex (Figure 4c,d,f; FC: 1.53) in 3xTgAD mice receiving the iPSC-NPC transplantation in comparison with the PBS-injected group (*n* = 7–8 mice/group, five sections per animal; *p* < 0.0001 and *p* < 0.05, respectively) (Figure 4e,f).

**Figure 5:**
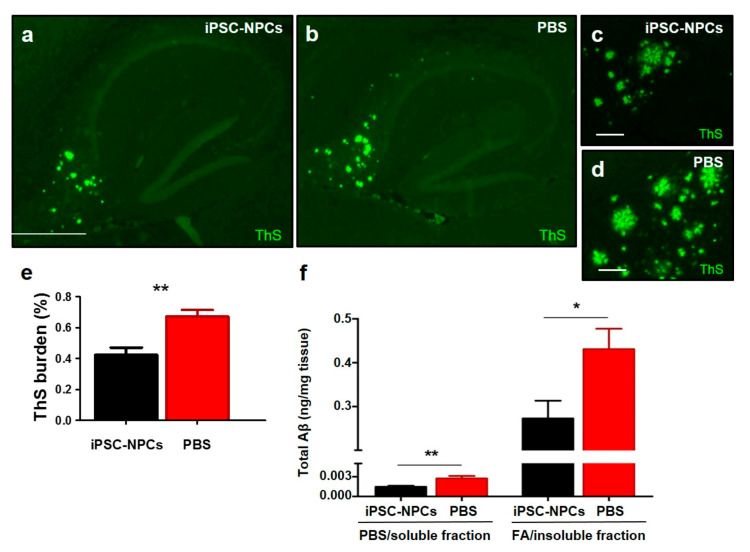
3xTg-AD mice transplanted with iPSC-NPCs displayed lower levels of ThS-positive amyloid deposits and soluble and insoluble Aβ in brains. (**a**–**d**) ThS-stained brain slices from 3xTg-AD iPSC-NPCs and PBS-injected mice were analyzed with an epifluorescent microscope. ThS-positive fibrillar deposits was reduced in the hippocampus of 3xTg-AD iPSC-NPCs-injected mice (**a**,**c**) compared with PBS-injected mice (**b**,**d**). Scale bar: (**a**,**b**) 300 μm; (**c**,**d**) 25 μm. (**e**) The burden of ThS-positive deposits was attenuated in 3xTg-AD iPSC-NPCs mice compared to 3xTg-AD PBS mice (student *t*-test, ** *p* < 0.01). (**f**) The quantity of PBS soluble and formic acid (FA) insoluble (aggregated) total Aβ was elevated in 3xTg-AD PBS mice compared to the iPSC-NPCs-injected counterpart as measured by ELISA (*n* = 7–8/group) (student *t*-test, ** *p* < 0.01; * *p* < 0.05). Data corresponds to means ± SEM.

Further evaluation of Aβ pathology in the hippocampus using ThS staining showed that the fibrillar Aβ load was also significantly reduced in 3xTg-AD iPSC-NPCs transplanted mice (Figure 5a–e; FC: 1.59; *p* < 0.01). In parallel, we performed biochemical measurements of soluble and insoluble Aβ. For this purpose, 10% *w/v* brain homogenate of each sample was prepared. ELISA quantification of samples fractionated by serial dilution in PBS and formic acid showed lower levels of soluble Aβ (*n* = 7–9 mice/group; FC: 1.40; *p* < 0.05) and insoluble Aβ (*n* = 7–9 mice/group; FC: 1.58; *p* < 0.05) in the brains of the 3xTgAD mice receiving the iPSC-NPC transplantation compared with their PBS-injected counterparts (Figure 5f). These results suggest that transplantation of iPSC-NPCs prevents the accumulation of Aβ and/or favors the clearance of Aβ deposits in the brain of 3xTg-AD mice.

**Figure 6:**
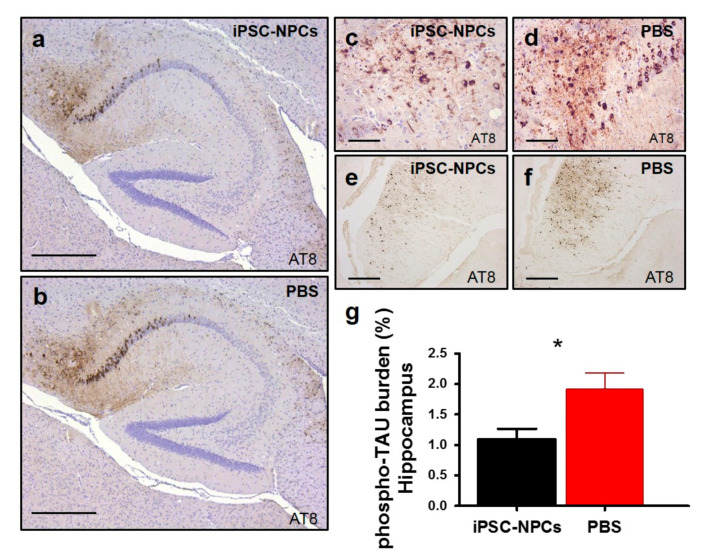
3xTg-AD mice transplanted with iPSC-NPCs show a reduction in tau pathology. We analyzed tau hyperphosphorylation by using a phospho-specific anti-tau antibody, AT8. Tau immunostaining was decreased in the hippocampus and fornix of 3xTg-AD mice injected with iPSC-NPCs (**a**,**c**,**e**) compared with PBS-injected 3xTg-AD mice (**b**,**d**,**f**). AT8 was counterstained with hematoxylin (**a**–**d**). Scale bar: (**a**,**b**) 300 μm; (**c**–**f**) 25 μm. (**g**) Image analysis of AT8 p-tau burden in the hippocampus revealed lowered levels in 3xTg iPSC-NPCs mice versus 3xTg-AD PBS mice (*n* = 7–8/group) (student *t*-test, * *p* < 0.05). Data corresponds to the means ± SEM.

Next, we wanted to study whether other hallmarks of AD pathology were influenced by the introduction of the iPSC-NPCs as observed for Aβ deposition. For this reason, we focused on analyzing alterations in p-tau and astrogliosis following an introduction of iPSC-NPCs in vivo. The disruption of normal phosphorylation of tau and a chronic inflammatory response are contributing factors to the pathogenic processes [41,42,43,44]. Representative immunohistochemical images utilizing the AT8 antibody (Figure 6a–f) and quantifications demonstrated that p-tau had overall decreased in the hippocampus and fornix in 3xTg-AD iPSC-NPCs mice compared to age-matched 3xTg-AD PBS mice (*n* = 7/group; FC: 1.76; *p* < 0.05) (Figure 6g).

**Figure 7:**
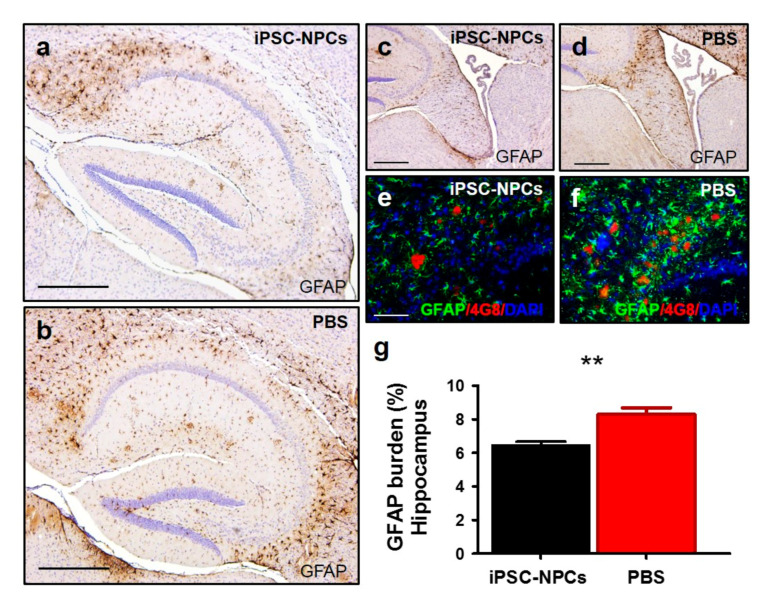
3xTg-AD mice transplanted with iPSC-NPCs show a reduction in astrogliosis. Astroglial cells were immunostained using the GFAP antibody, which recognizes an intermediate filament protein (glial fibrillary acidic protein) expressed mainly in astrocytes. GFAP immunostaining was decreased in 3xTg-AD mice injected with iPSC-NPCs (**a**,**c**,**e**) compared with PBS-injected 3xTg-AD mice (**b**,**d**,**f**). Light microscopic images of GFAP-immunoreactivity shown in the hippocampus (**a**,**b**,**e**,**f**) and fornix (**c**,**d**). GFAP was counterstained with hematoxylin (**a**–**d**). Slides were also co-stained with GFAP (green), 4G8 (red), and DAPI (blue) (**e**,**f**) to label astrogliosis, amyloid plaques, and nuclei, respectively and analyzed with an epifluorescent microscope. Scale bar: (**a**,**b**) 300 μm; (**c**,**d**) 100 μm; (**e**,**f**) 25 μm. (**g**) Quantification of the astrocytes-stained area demonstrated a reduced GFAP burden in the hippocampus of 3xTg-AD iPSC-NPCs mice compared to 3xTg-AD PBS mice (*n* = 7–8/group) (student *t*-test, ** *p* < 0.01). Data corresponds to the means ± SEM.

Reactive astrocyte levels analyzed by GFAP staining were also reduced in the hippocampus and fornix of 3xTg-AD mice receiving iPSC-NPC injection in comparison with PBS-injected animals (Figure 7a–f). Quantification of GFAP in the hippocampus revealed that there was a 1.29 FC reduction in astrocyte reactivity in aged 3xTg-AD iPSC-NPCs treated mice compared to 3xTg-AD PBS animals (*n* = 7–8/group; Figure 7g; *p* < 0.01). Furthermore, we noticed a reduction in the cell body hypertrophy and thickening of the processes of reactive astrocytes in the iPSC-NPCs transplanted group (Figure 7e,f). At this point, the exact mechanism by which the administration of iPSC-NPCs led to a reduction in the accumulation of Aβ and p-tau and a decrease of brain astrogliosis remains to be elucidated however, future studies are ongoing to further study this.

## 4. Discussion

Neurodegenerative diseases are a global problem affecting the elderly population worldwide. The central and shared characteristics of neurodegenerative diseases are the presence of insoluble protein aggregates and the loss of neuronal cells and synaptic connections, which lead to clinical symptoms. AD is the most common neurodegenerative disease, manifested by chronic and progressive memory and intellectual decline [2]. In spite of extensive effort to develop a treatment against AD, there are no effective therapies currently available. The existing therapeutic interventions, such as cholinesterase inhibitors, have only a very modest effect in alleviating the symptoms of AD and do not alter the course of the disease [4,45]. Unfortunately, in recent years many clinical trials with drugs targeting the cellular pathways that are thought to be key for the disease, have consistently failed [5]. The difficulties for developing effective therapies are likely due to the progressive nature of the disease, the incomplete understanding of its molecular basis, the limited regenerative capacity of the brain, and the lack of an early diagnosis. At this time, patients are diagnosed by the appearance of clinical symptoms, which are evident only after extensive brain damage [4]. As a result, novel approaches are urgently needed to slow down or stop the disease progression or even to recover some of the lost brain function. Considering that AD is characterized by the massive loss and dysfunction of neurons in the brain, cell-replacement therapies, such as iPSC-derived brain cells, hold a great potential for treating AD patients who may be beyond the help of more classical pharmacological therapies [46].

In the present study, we analyzed the therapeutic effect of intracerebral injection of iPSC-NPCs to rescue brain impairment in an AD animal model at a late stage in the disease as an indicator of its potential to improve cognitive and neurological function in humans with AD. For this purpose, we selected the 3xTg-AD mice model, which overexpress mutated forms of three human genes linked to AD (APP_SWE_, PS1_M146V_, and Tau_301L_) and develop an age-dependent pathology similar to AD brains, including the accumulation of amyloid plaques and NFTs as well as some markers of neurodegeneration and some clinical phenotype including memory dysfunction [27,47]. The specific role of amyloid and NFTs in AD is not completely understood, but several evidences suggest that tau pathology may be more important for neurodegeneration than amyloid plaques [43]. Thus, we consider this animal to mimic more closely AD pathogenesis than the models using only APP mutations. Our strategy involved the bilateral hippocampal injection of mouse iPSC-NPCs in the 3xTg-AD transgenic mouse model. Animals were treated at 17 months, an age in which these mice have severe behavioral deficits, synaptic impairment, and typical AD pathological abnormalities [27,28,47]. We believe that an invasive therapy such as intra-cerebral injection of neuronal precursors derived from stem cells can only be applied to more advanced cases of the disease where non-invasive treatments available do not produce benefit. Furthermore, it is very important to develop a treatment for severe cases of the disease and we believe that regenerative therapies offer the best hope for these cases. Strikingly, our study revealed that the transplantation of iPSC-NPCs improved AD-like phenotypes, including memory and learning, synaptic plasticity, and decreased deposition of Aβ and p-tau aggregates and brain astrogliosis.

Spatial learning and memory were assessed using behavioral tests specific for medial temporal lobe function—Barnes Maze and OLT. Before the injection of the cells, 3xTg-AD mice had impaired spatial performance measured by OLT at 17 months. However, after iPSC-NPCs transplantation, 3xTg-AD mice displayed significantly improved OLT performance even to similar levels as WT iPSC-NPCs and PBS controls. This improvement was not only seen post-inoculation among the groups, but also an analysis of individual animals before and after treatment showed a clear reversal of impaired spatial abilities. The increase in performance following injection was not observed in the control 3xTg-AD PBS mice, which eliminates the possibility of being an effect of the surgery. Moreover, results posit no deleterious effect of transplanted iPSC-NPCs in animals. In the Barnes maze paradigm, 3xTg-AD iPSC-NPCs-treated animals found the escape location faster than 3xTg-AD PBS-injected mice during the training phase indicating that this group of animals has a better ability to learn the task. In addition, 3xTg-AD iPSC-NPCs-transplanted mice exhibited a comparable performance to WT iPSC-NPCs transplanted mice and WT PBS-injected mice controls 7 days after the final training trial (long-term memory). These results suggest that the treatment produced a substantial recovery in spatial memory.

The deep memory deficits in AD patients may be caused by alterations in synaptic plasticity which has been supported by previous studies in transgenic mouse models [27,28,48]. Therefore, we assessed whether the improvement in hippocampal-dependent memory observed in 3xTg-AD treated with iPSC-NPCs correlates with the ability to induce LTP in the CA1-CA3 connections. We used two types of stimulation protocols widely utilized to induce LTP by delivering a weak or strong stimulation (see Methods). Interestingly, a weak TBS protocol failed to potentiate synapses of PBS-injected 3xTg-AD mice but successfully induced potentiation in synapses from 3xTg-AD iPSC-NPCs mice. It is known that the experimental induction of LTP in a population of synapses can be more effective if baseline transmission is higher, as this facilitates postsynaptic depolarization [49]. However, since basal synaptic transmission in iPSC-NPCs-treated transgenic mice proved to be lower than for PBS-injected mice, LTP enhancement cannot be attributed to this factor, instead reflecting the strengthening of the mechanism underlying this form of synaptic plasticity. The subsequent application of a strong—presumably saturating protocol—induced LTP in both groups. Interestingly, the maximal percent potentiation relative to baseline was similar in both cases. This suggests that the threshold for LTP induction was modified, favoring the generation of this form of synaptic plasticity in the iPSC-NPCs-treated 3xTg-AD mice. A lower threshold for LTP induction may permit regular environmental stimuli (as those used in our behavioral experiments) to be learned more efficiently. In addition, the lack of change in paired-pulse facilitation suggests that the lower basal synaptic strength in 3xTg-AD iPSC-NPCs mice relative to PBS-injected controls is not due to a change in neurotransmitter release probability. Instead, a decrease in the number of functional connections or a down-regulation in synaptic AMPA-receptors may underlie this difference. The decrease in basal transmission after iPSC-NPCs-transplantation, accompanied by the shift in the threshold for LTP to lower values, a phenomenon called metaplasticity, suggests the possibility that the treatment is restoring homeostatic processes that normally occur in neuronal networks, which may be altered in old 3xTg-AD mice. These homeostatic changes allow for maintaining synaptic function in a dynamic range by adjusting synaptic strength and modifying the threshold for plasticity according to circuit activity [50]. Such mechanisms may permit further activity-dependent synaptic enhancement thereby avoiding plasticity saturation. Interestingly, recent evidence from animal models and human AD patients supports the involvement of altered mechanisms of homeostatic metaplasticity in the pathogenesis of AD [51,52].

An additional point to be considered here are the fundamental differences between TBS and HFS protocols regarding their real contribution to synaptic plasticity in the intact brain. While both strategies effectively induce LTP in experimental conditions, there seem to be important differences in their functional relevance: TBS mimics activity patterns observed in the hippocampus in vivo during spatial learning, whereas HFS does not resemble neuronal activity in physiological conditions [53]. Considering this evidence, it is reasonable to speculate that alterations in TBS-induced potentiation, as those reported here, may underlie at least in part, the memory deficits detected in PBS-injected 3xTg-AD mice compared to those with iPSC-NPCs-transplanted 3xTg-AD mice. Therefore, the finding that both LTP and learning skills are improved by iPSC-NPCs transplantation points to this therapy as a powerful tool in the treatment of cognitive damage in AD.

The cause of synaptic impairment and memory dysfunction in AD is currently unknown, but many genetic, cell biology, and biochemical studies suggest that the accumulation of protein aggregates might be the triggering event in the neurodegenerative cascade [54,55]. The accumulation of misfolded protein aggregates in the form of extracellular amyloid plaques and intracellular NFTs are the pathognomonic features of AD [2,54]. In addition to the improvements in memory and brain functionality, 3xTg-AD treated with iPSC-NPCs demonstrated a highly significant decrease of Aβ plaque formation and reduced levels of p-tau. Treated animals also exhibited lower levels of astrogliosis, which is another typical feature of AD that is thought to be involved in neurodegeneration [41,56].

Replacement of dysfunctional neurons is often seen as the primary mechanism by which a stem cell therapy may produce benefit [57,58,59]. However, in our study it is not clear whether the beneficial effects produced by iPSC-NPCs transplantation in 3xTg-AD mice are due to the differentiation, maturation, and/or integration of the transplanted cells. Few iPSC-NPCs were able to survive (~10% of the injected cells), incorporate, and differentiate into neurons, astrocytes, and oligodendrocytes in the hippocampus of 3xTg-AD mice after 2 months. Similar observations have been reported by other researchers using other cell types showing that only a small proportion of transplanted cells survive and engraft into injured tissues [33,34,35]. For example, human mesenchymal stem cells unilaterally implanted into the dentate gyrus of the hippocampus of adult immuno-deficient mice did not proliferate, and no more than 26% of these cells survived for even 3 days after the implant. Instead, the implanted mesenchymal stem cells stimulated the proliferation of endogenous NSCs within the hippocampus [35]. Thus, it is possible that the improvement in AD pathology observed in our study could be attributed, instead of cell replacement, to the beneficial effects from alternative/indirect mechanisms associated with the transplanted cells, often referred to as a “bystander effect” [48]. Recently, evidence has emerged that the transplantation of stem cells into brains with neurodegenerative diseases actually promotes brain repair via trophic mechanisms resulting from the release of bioactive factors (i.e., cytokines, chemokines, and neurotrophins) and modulating the immune responses even after transplanted cells die. Interestingly, NSCs can express high levels of neurotrophins, including brain-derived neurotrophic factor (BDNF) and nerve growth factor (NGF) [36,60,61]. For example, it has been shown that NSC grafts increase BDNF levels and lead to behavioral rescue without changing Aβ or tau pathologies in the 3xTg-AD mouse model [36]. It seems that the secretion of this trophic factor from the transplanted NSCs is required for rescuing the cognitive function in AD transgenic mice since shRNA-mediated BDNF knockdown abolishes this rescue [36]. Other stem cell populations, including MSCs and ESCs, can also produce several neurotrophins [62,63]. Thus, stem cells could provide a means to deliver neurotrophins to the diseased brain, potentially modulating endogenous synaptic plasticity, enhancing neuronal survival, and improving memory and learning. Supporting this conclusion, in vitro assays showed that our iPSC-NPCs were able to release BDNF into the culture medium.

Additionally, aged 3xTg-AD iPSC-NPCs mice exhibited diminished astrocytosis in the hippocampus as compared to aged 3xTg-AD PBS mice. Glial cells are a key player in the inflammatory response in disease as post-mortem brains of AD patients and in AD animal models displaying deleterious neuroinflammation. Moreover, astrocyte exacerbation may occur early in AD, even before Aβ deposition, and GFAP-positive astrocytes can accumulate around senile plaques [9]. Astrocytes can release cytokines, interleukins, nitric oxide, and other possible cytotoxic molecules after exposure to Aβ, thereby unknowingly exacerbating the neuroinflammatory response [41,56]. Recently, we had performed the intravenous delivery of NPCs in a Parkinson’s disease mouse model, and the NPCs treatment ameliorated motor function and attenuated the neuroinflammatory response [18]. Moreover, previous reports suggest the activation of microglia, recruitment of microglia to amyloid plaques for clearance, and increased expression of Aβ degrading enzymes as additional molecular mechanisms [6,7,8]. Thus, a similar effect could be occurring here as reducing neuroinflammation presents a beneficial response in the 3xTg-AD mouse model.

Obviously, there are several potential mechanisms by which NPCs may produce a beneficial outcome, including the increase of neurogenesis, protein homeostasis, degradation of misfolded proteins, reduction of brain inflammation, etc. Regardless of the exact mechanism by which iPSC-NPCs improve brain abnormalities, memory, and synaptic properties in vivo, our results, along with others, support stem cell therapy using iPSC-NPCs as a promising approach for aged individuals already clinically diagnosed and/or in later stages of insidious neurodegenerative diseases, such as AD. Future studies look to explore a definitive molecular mechanism of the beneficial effects of iPSC-NPCs treatment, and since AD is an age-related, chronic malady, there is interest in investigating the effect, amount, and how long iPSC-NPCs can survive and/or integrate in an age-dependent manner.

## Data Availability

Data available on request to the corresponding author.

## Supplementary Materials

The following are available online at https://www.mdpi.com/article/10.3390/cells10071802/s1, Table S1: Primer sequences for mouse ESC genes, Figure S1: Generation of iPSCs from adult mouse tail-tip fibroblasts and analysis of pluripotency, Figure S2: Derivation of mouse NPCs from iPSCs (iPSC-NPCs). Figure S3: In vitro differentiation of iPSC-NPCs, Figure S4: Induction of GFP expression in iPSC-NPCs.
