# Supplementary material for: Induced Pluripotent Stem Cell-Derived Neural Precursors Improve Memory, Synaptic and Pathological Abnormalities in a Mouse Model of Alzheimer’s Disease
Source: Cells. 2021 Jul 16;10(7):1802. doi: 10.3390/cells10071802 (PMC8303262; doi:10.3390/cells10071802)

SUPPLEMENTARY FIGURE LEGENDS

Supplementary Figure 1. Generation and characterization of iPSCs from adult mouse tail-tip fibroblasts (TTFs). (**a**) Schematic representation of the experimental design to generate iPSCs. Mouse TTF were transduced one time (day 0 post transduced, 0 DPT) with four PMXs retroviral reprogramming plasmids (Addgene). Six DPT, mouse TFFs were collected and seeded with irradiated MEFs. Mouse TTFs medium was replaced with mouse ESCs medium every day. At 30 DPT, mouse ESC-like colonies were detached from the plate using a rounded tip glass Pasteur pipette, collected and disaggregated manually. (**b**) Morphology of established iPSC line miPSC-TTF1 at passage number 10. miPSC-TTF1 line was positive for alkaline phosphatase (**c**) and the pluripotent markers SSEA-1 (**d**), OCT4 (also known as *Pou5f1*) (**d**), E-cadherin (**e**) and SOX2 (**e**). Nuclei were stained with DAPI (blue). Scale bar (b,d,e): 25 μm, (c): 50 μm. (**f**) RT-PCR showed miPSC-TFF-1 and miPSC-TFF-2 lines expressed many undifferentiated ESC marker genes. (**g-l**) Pluripotency of iPSCs derived from adult TTPs *in vitro*. miPSC-TTF-1 colony was harvested by trypsinization and plated on low-attachment plates in the mouse ESC medium without LIF. After 3 days, aggregated cells (**g**) were transferred onto gelatin-coated tissue culture dishes and incubated for one week (**h**). Scale bar (g,h): 50 μm. Cells were then fixed and incubated with anti-α-fetoprotein (AFP) (**i**), anti-brachyury (**j**) or anti-microtubule-associated protein 2 (MAP2) (**k**) antibodies. Signals were visualized with Alexa Fluor 488 secondary antibody. Nucleus were stained with DAPI. Scale bar (i-k): 50 μm. (**l**) We performed RT-PCR with total RNA isolated from EB miPSC (EBmiPSC-TTF-1), EB mouse ESCs, mouse ESCs and MEFs. The expression of Brachyury (mesoderm marker), Gata6 (endoderm marker) and Map2 (ectoderm marker) were examined. Nat1 was used as a loading control.

Supplementary Figure 2. Derivation and characterization of mouse iPSC-NPCs. (**a**) iPSC-NPCs have elongated bipolar morphology, end-feet and oval nucleus. (**b-e**) iPSC-NPCs were positive for markers typically observed in authentic NPCs, including nestin, vimentin, musashi and Sox2. (**f, g**) Some iPSC-NPCs also express the neuron-restricted markers doublecortin (DCX) and MAP2, the astrocyte marker (GFAP) (**h**) and the oligodendrocyte marker (OLIG1) (**i**). (**j**) Pluripotent markers Oct4 and SSEA-1 were silenced in iPSC-NPCs. Scale bar (a-j): 25 μm**.** (**k**) Flow cytometric analysis of iPSC-NPCs showing expression of Nestin, E-Cadherin, DCX, and GFAP, but not Oct4.

Supplementary Figure 3. ***In vitro*** **differentiation of iPSC-NPCs.** *In vitro* differentiation showed that iPSC-NPCs were able to generate neurons (**a**), astrocytes (**b**) and oligodendrocytes (**c**) after 3 weeks. Scale bar: 25 μm

Supplementary Figure 4. Induction of GFP expression in iPSC-NPCs. iPSC-NPCs were transduced with rtTA and Dox-GFP lentivirus. iPSC-NPCs GFP^+ were then sorted using FACS and expanded. Isolated cells were cultured in the absence (**a**) or presence (**b**) of dox (1 g/ml) for 24 h. Dishes were photographed using a fluorescence microscope. Cll sortin studies showed that 98.75% of the cells were positive for GFP after dox treatment. Scale bar: 25 μm

Supplementary Table 1. Primer sequences for mouse ESC genes

Symbol	Primer sequence 5' to 3'	Gene
Ecat1-RT-S	TGT GGG GCC CTG AAA GGC GAG CTG AGA T	RT-PCR for Ecat1
Ecat1-RT-AS	ATG GGC CGC CAT ACG ACG ACG CTC AAC T	
pH34-U38	GAA GTC TGG TTC CTT GGC AGG ATG	RT-PCR for Esg1
pH34-L394	ACT CGA TAC ACT GGC CTA GC	
6047-S1	CAG GTG TTT GAG GGT AGC TC	RT-PCR for endogenous Nanog
6047-AS1	CGG TTC ATC ATG GTA CAG TC	
45328-S118	ACT GCC CCT CAT CAG ACT GCT ACT	RT-PCR for ERas
ERas-AS304	CAC TGC CTT GTA CTC GGG TAG CTG	
Gdf3-U253	GTT CCA ACC TGT GCC TCG CGT CTT	RT-PCR for Gdf3
Gdf3-L16914	AGC GAG GCA TGG AGA GAG CGG AGC AG	
Oct3/4-S9	TCT TTC CAC CAG GCC CCC GGC TC	RT-PCR for endogenous Oct3/4
Oct3/4-AS210	TGC GGG CGG ACA TGG GGA GAT CC	
Rex1-RT-S	ACG AGT GGC AGT TTC TTC TTG GGA	RT-PCR for Rex1
Rex1-RT-AS	TAT GAC TCA CTT CCA GGG GGC ACT	
Utf1-RT-S	GGA TGT CCC GGT GAC TAC GTC TG	RT-PCR for Utf1
Utf1-RT-AS	GGC GGA TCT GGT TAT CGA AGG GT	
Zfp296-S67	CCA TTA GGG GCC ATC ATC GCT TTC	RT-PCR for Zfp296
Zfp296-AS350	CAC TGC TCA CTG GAG GGG GCT TGC	
Nat1-U283	ATT CTT CGT TGT CAA GCC GCC AAA GTG GAG	RT-PCR for Nat1
Nat1-L476	AGT TGT TTG CTG CGG AGT TGT CAT CTC GTC	
Sox2-RT-S	TAG AGC TAG ACT CCG GGC GAT GA	RT-PCR for endogenous Sox2
Sox2-RT-AS	TTG CCT TAA ACA AGA CCA CGA AA	
Klf4-S1236	GCG AAC TCA CAC AGG CGA GAA ACC	RT-PCR for endogenous Klf4
Klf4cDNAas2547	TCG CTT CCT CTT CCT CCG ACA CA	
Myc-S1904	TGA CCT AAC TCG AGG AGG AGC TGG AAT C	RT-PCR for endogenous c-Myc
Myc-AS2042	AAG TTT GAG GCA GTT AAA ATT ATG GCT GAA GC	
Mtap2-S629	CAT CGC CAG CCT CGG AAC AAA CAG	RT-PCR for Map2
Mtap2-AS867	TGC GCA AAT GGA ACT GGA GGC AAC	
T-S764	ATG CCA AAG AAA GAA ACG AC	RT-PCR for Brachyury
T-AS1579	AGA GGC TGT AGA ACA TGA TT	
Gata6-S917	ACC TTA TGG CGT AGA AAT GCT GAG GGT G	RT-PCR for Gata6
Gata6-AS1250	CTG AAT ACT TGA GGT CAC TGT TCT CGG G	
MeOct3/4-DMR-S	GGT TTT TTA GAG GAT GGT TGA GTG	Methylation analysis of Oct3/4
MeOct3/4-DMR-AS	TCC AAC CCT ACT AAC CCA TCA CC	
MeNanog-F2-S	GAT TTT GTA GGT GGG ATT AAT TGT GAA TTT	Methylation analysis of Nanog
MeNanog-F2-AS	ACC AAA AAA ACC CAC ACT CAT ATC AAT ATA	

Supplementary Figure 1

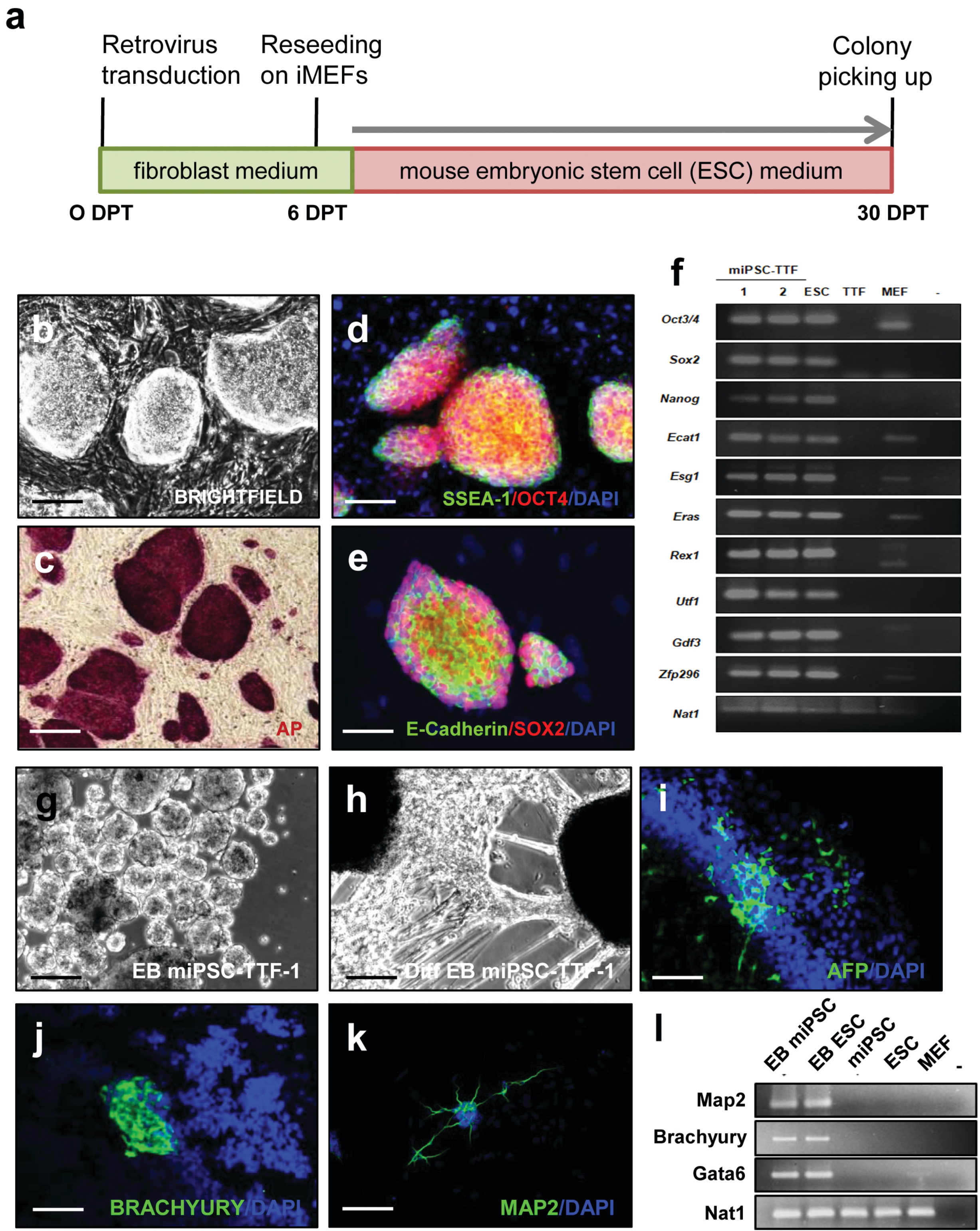

Supplementary Figure 2

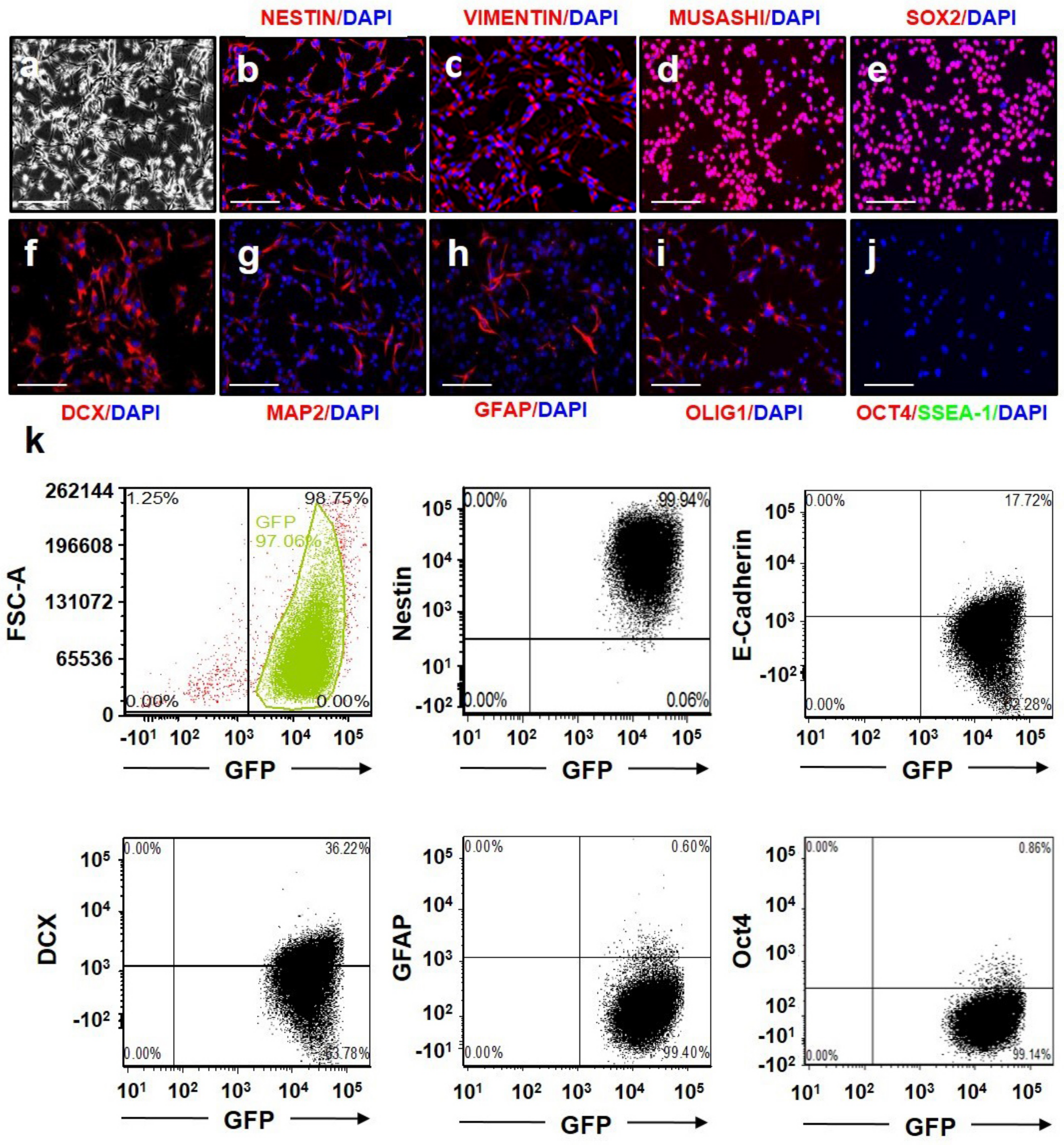

Supplementary Figure 3

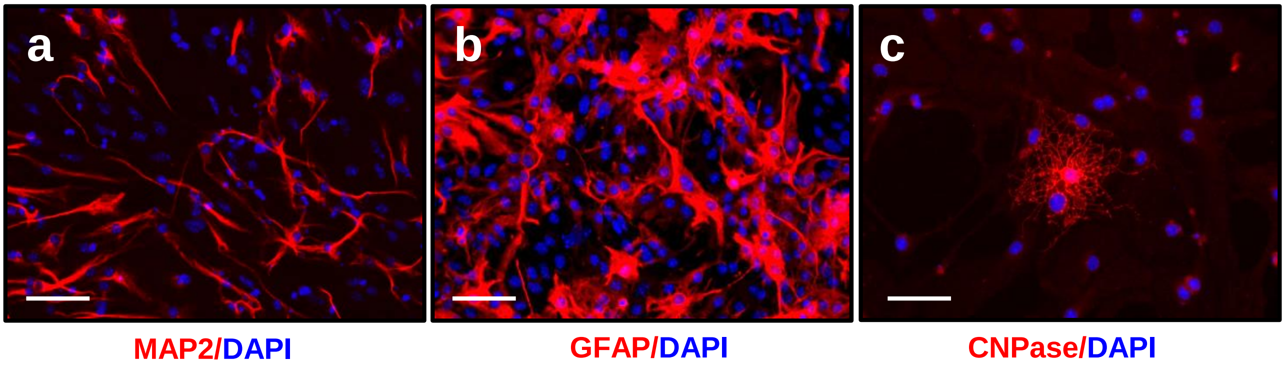

Supplementary Figure 4

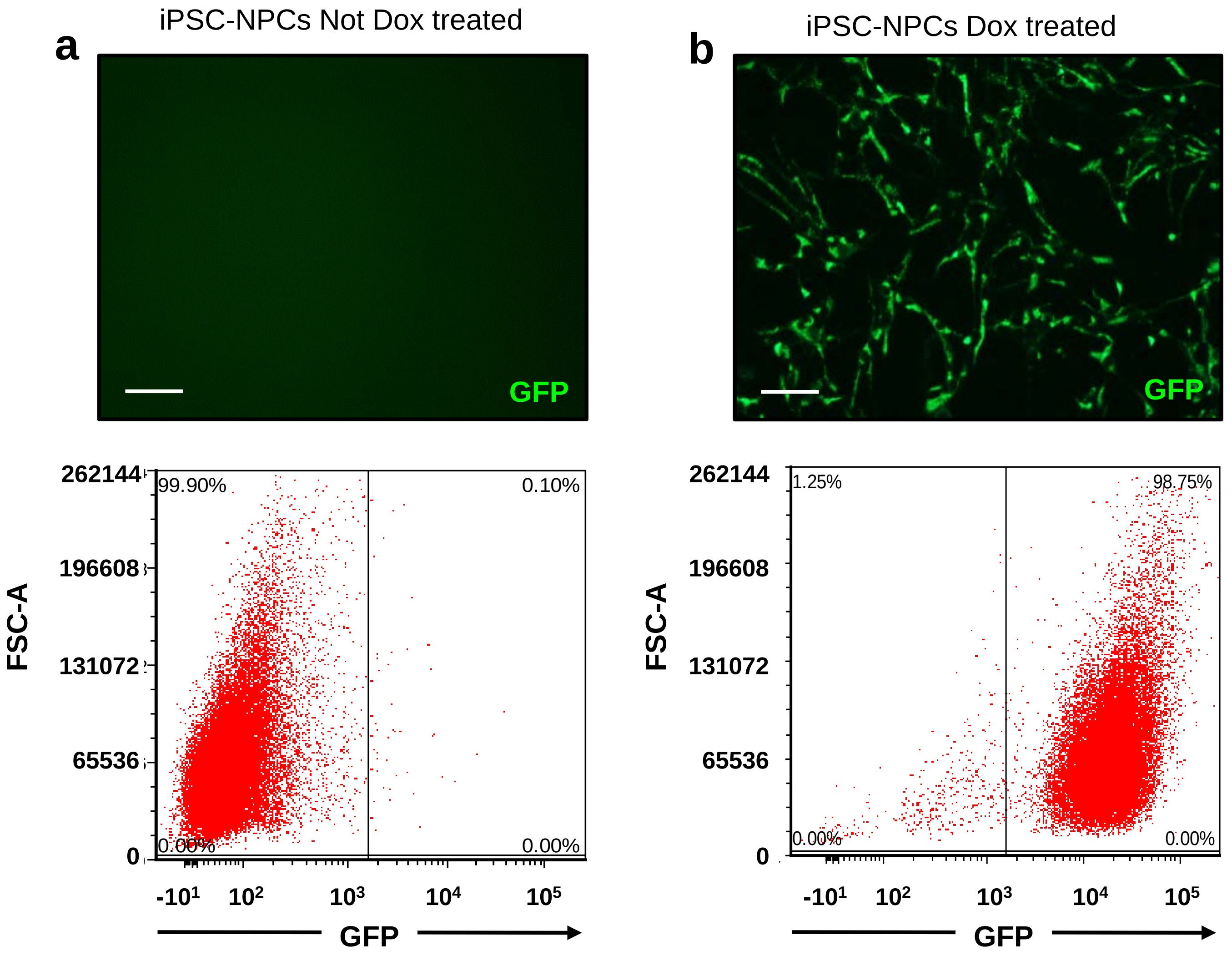

Supplement: Supplementary file 1 [file cells-10-01802-s001.zip › cells-1253073-supplementary.pdf]
